# Bioleaching of uranium from ores and rocks using filamentous fungi

**DOI:** 10.3389/fmicb.2025.1523962

**Published:** 2025-04-30

**Authors:** Tariq M. Bhatti, Olli H. Tuovinen

**Affiliations:** ^1^Department of Chemical Engineering, Pakistan Institute of Engineering and Applied Sciences, Islamabad, Pakistan; ^2^Department of Microbiology, The Ohio State University, Columbus, OH, United States

**Keywords:** *Aspergillus*, carboxylic acids, citric acid, *Penicillium*, uranium biorecovery

## Abstract

The purpose of this article is to review the role of filamentous fungi in the leaching of uranium from ores and rocks. Fungi produce short-chain fatty acids through the fermentation and partial oxidation of organic compounds. Biomass can be separated from the culture, while organic acids in spent media dissolve and sequester uranium from minerals in ores and rocks. Oxide, carbonate, and silicate ores containing hexavalent uranium are suitable for this treatment. Fungi can maximize the production of short-chain fatty acids. Uranium dissolution and sequestration are attributed mostly to the formation of soluble U(VI)-carboxylate complexes with citric and oxalic acids, although other carboxylic acids can also sequester uranium. The leach solutions maintain a pH level between 1.5 and 3.5, as the low pH facilitates proton attack on the minerals and minimizes the precipitation of metals in the leach solution. Two types of uranium leaching processes using fungi have been tested: one-step leaching, where biomass is not separated before contact with the uranium mineral, and two-step leaching, which employs spent medium after the removal of fungal biomass. Process optimization to the pilot stage has not yet been reported in the literature. To date, this article is the first to review the role of filamentous fungi in uranium bioleaching from ores and rocks.

## 1 Introduction

Several applications of heterotrophic microorganisms for metal recovery are under investigation. Filamentous fungi and other heterotrophs produce low molecular weight carboxylic acids, which can be used as leach solutions for the dissolution of metals from ores and e-waste. Fungal species and genera vary in the types and concentrations of organic acids they produce. Carboxylic acids produced by *Aspergillus* spp. and other filamentous fungi have been tested as leach solutions for extracting metals from spent batteries (Kim et al., 2016; Jadhav et al., 2018). Printed circuit boards found in electronic waste also present potential sources of metals that can be recovered using organic acids produced by heterotrophs (Chauhan et al., 2018; Argumedo-Delira et al., 2019; Dutta et al., 2023; Moosakazemi et al., 2023). Additional resources for heterotrophic bioleaching include municipal solid waste, industrial sludges, and various solid and liquid waste streams. Many of these promising bioleaching processes have been reviewed in recent literature (Chauhan et al., 2018; Baniasadi et al., 2019; Islam et al., 2020; Kara et al., 2023).

Bioleaching is also a viable option for low-grade uranium ores that are difficult or uneconomical to process using conventional hydrometallurgy. Uranium is a naturally occurring radioactive element in the actinide group of the periodic table. The most common isotopes include ^92^U^238^, ^92^U^235^, and ^92^U^234^. The most abundant isotope, which makes up 99.28% of the natural isotopic composition of uranium, is ^92^U^238^. This isotope decays 14 times through alpha or beta emission before reaching stable lead (^82^Pb^206^), with a half-life of 4.468 billion years.

The mineralogy and concentration of uranium in rocks and ores vary considerably. Uranium mineralizations are found in igneous, metamorphic, and sedimentary rock types worldwide (Dahlkamp, 1993; Hore-Lacy, 2016; International Atomic Energy Agency, 2018). Approximately 300 uranium minerals have been identified in various forms, including halides, oxides, carbonates, sulfates, phosphates, arsenates, vanadates, and silicates (International Atomic Energy Agency, 2020; Skirrow et al., 2009). The primary uranium minerals contain U(IV) and are found in their original state in vein deposits or pegmatites. Secondary uranium minerals are altered, usually due to chemical weathering, and contain U(VI) or a combination of reduced and oxidized uranium. Uranium also occurs in organic complexes such as thucholite and uranyl oxalate minerals, including uroxcite and metauroxcite (Kampf et al., 2020). Approximately 30% of uranium reserves are located in sandstone deposits, typically exhibiting mixtures of U(IV) and U(VI) mineralizations due to past redox and anoxic/oxic sedimentary conditions (Jin et al., 2020).

The role of acidophilic iron-and sulfur-oxidizing autotrophic bacteria is well-established in the bioleaching of uranium and sulfide ores in the mining industry (Kaksonen et al., 2020). These bioleaching processes can treat multi-metal ore materials and recover transition metals or rare earth elements, thereby enhancing economic efficiency. Filamentous fungi can also significantly contribute to bioleaching processes for recovering uranium and other metals. Optimizing the cultivation of fungi can result in high concentrations of organic acids, which dissolve and sequester metals from minerals. Citric acid and oxalic acid produced in cultures of *Aspergillus* spp. and other filamentous fungi form soluble complexes with uranium and transition metals upon their dissolution at acidic pH when interacting with ore material (Dusengemungu et al., 2021). The sequestration and acid solution chemistry effectively reduce the precipitation of dissolved metals as hydroxides, carbonates, or other poorly soluble complexes.

Fermentation is the primary method of organic acid production by filamentous fungi. They produce mixtures of carboxylic acids that vary in composition and concentration (Liaud et al., 2014; Yang et al., 2017). These acids include acetic acid (linear formula CH_3_COOH), citric acid (HOC(COOH)(CH_2_COOH)_2_), fumaric acid (HOOCCH=CHCOOH), gluconic acid (HOCH_2_(CHOH)_4_COOH), ketoglutaric acid (HOOCCH_2_CH_2_COCOOH), malic acid (HOOCCH_2_CH(OH)COOH), maleic acid (HOOCCH=CHCOOH), oxalic acid (HOOCCOOH), succinic acid (HOOCCH_2_CH_2_COOH), and tartaric acid (HOOCCH(OH)CH(OH)COOH). Citric acid and oxalic acid are often the primary short-chain fatty acids found in culture solutions produced by filamentous fungi.

The purpose of this review is to examine the applications of filamentous fungi for leaching uranium from rocks and ores. Filamentous fungi require organic carbon sources, such as monosaccharides or some disaccharides, for growth and energy generation. Certain agricultural waste materials, including tea waste and wheat bran (Ahmed et al., 2024), can also serve as carbon sources; however, sterilization or at least pasteurization may be necessary to eliminate potential overgrowth by native microorganisms. Fungi do not gain direct metabolic benefits from the dissolution and degradation of minerals. Filamentous fungi are ubiquitous in both pristine and engineered environments, and the production of organic acids is a relatively common trait among them. This organic acid production plays an important ecological role in biogeochemical processes, as it affects the corrosion of metallic structures and the solubilization of metals and metalloids in soils, waste disposal sites, and other geochemical contexts.

## 2 Bioleaching of uranium by filamentous fungi: background

Filamentous fungi solubilize uranium and other metals from ores and rocks through the formation of carboxylic acids, which mediate acid attack (acidolysis) and create soluble metal complexes (complexolysis) during the bioleaching process. Among filamentous fungi, *Aspergillus* and *Penicillium* spp. have been primarily tested for applications in fungal bioleaching. The list in Table 1 presents potential targets for bioleaching applications, as examined in recent literature, using carboxylic acids produced by *Aspergillus* species and other filamentous fungal genera. Carboxylic acids are recognized as effective lixiviants for the dissolution of metals from ores and industrial e-waste streams. However, a concise review of published studies on the bioleaching of uranium from ores and rocks using fungi and carboxylic acids has not been available. This article is the first to comprehensively review uranium bioleaching from ores by filamentous fungi.

**Table 1:** Selected examples of recent experimental fungal studies and reviews on the leaching metals from waste materials.

Waste material	Target metals	Fungal genus	Reference
Solid waste materials and low-grade ores and tailings	Various metals and metalloids	*Aspergillus, Penicillium, Cladosporium*	Dusengemungu et al. (2021)
Spent automotive catalytic converters	Rare earth elements	*Aspergillus*	Bahaloo-Horeh and Mousavi (2022)
Spent Li coin cells	Li, Mn	*Aspergillus*	Naseri et al. (2023)
Mine tailings	Mn, Ag	*Aspergillus, Penicillium*	Dong et al. (2023)
Mobile phone scrap	Cr, Zn, Pb	*Aspergillus*	Al-Shammari et al. (2023)
Solid waste	Ni, V, Al, Sb, Mo, Co, W, Cd, Cu, As, Cr, Pb, Ag, Au, Mn, Pd	*Aspergillus, Penicillium, Geotrichum, Fibroporia*	Rendón-Castrillón et al. (2023)
Coal ash	Rare earth elements	*Aspergillus*	Ma et al. (2023)
Spent lithium batteries	Li	*Aspergillus, Penicillium*	Kazemian et al. (2023)
Zinc purification residue	Zn, Co, Mn	*Aspergillus*	Faraji et al. (2023)
Rare earth ore	Rare earth elements	*Aspergillus*	Zhou et al. (2023)
Spent batteries	Li, Co, Ni, Mn, Cu, Zn	*Aspergillus*	Moosakazemi et al. (2023)
E-Waste	In, Cu, Ni	*Aspergillus*	Dutta et al. (2023)
Lithium batteries	Li, Co, Ni, Mn	*Aspergillus, Penicillium*	Roy et al. (2023)
E-waste	Li	*Aspergillus, Penicillium*	Priyadarsini and Das (2025)
Ores	Zn, Au, Ag,	Basidiomycota	Hein et al. (2023)
Printed circuit boards of mobile phones	Cu, Ni, Zn	*Aspergillus*	Trivedi and Hait (2024)
Electronic waste	Mostly Fe, Co, Cu, Ni, Zn	*Aspergillus*, *Penicillium*	Jaiswal and Srivastava (2024)
Liquid crystal displays	As, Al, In, Sr	*Aspergillus*	Parsa et al. (2024)
Power plant ash, spent refinery catalyst, low-grade ores, tailings	Ni, Mn	*Aspergillus, Penicillium*	Kara et al. (2023)
Uranium ores and rocks	U	*Aspergillus*	This review

The literature cited in these publications is extensive on potential fungal leaching of base metals and rare earth elements.

Filamentous fungi (e.g., species from the genera *Aspergillus*, *Cladosporium*, *Coniochaeta*, *Penicillium*, and *Talaromyces*) have been readily enriched and isolated from water and raffinate samples at uranium mines in various locations (e.g., Mishra et al., 2009; Vázquez-Campos et al., 2014; Coelho et al., 2020a). Fungi, including arbuscular mycorrhizae, are also nearly ubiquitous in other metal and coal mining sites (Ganesan et al., 1991; Glukhova et al., 2018; Wu et al., 2022; Kasatkina et al., 2023). Media for fungal cultivation typically rely on sucrose (Czapek’s broth) or glucose for energy, alongside mineral salts such as inorganic phosphate, a nitrogen source, Mg, Ca, and trace metals. Yeast extract has been incorporated into some formulations to supply additional trace cofactors. Complex, undefined media such as Sabouraud’s dextrose broth and potato dextrose broth have also been used for the cultivation of fungi in biomass experiments. The initial pH is commonly circumneutral but decreases due to the production of organic acids. Some cultivation methods employ pH stat systems to maintain pH within the neutral range since increasingly acidic values can inhibit fungal culture growth. Most fungi that have been tested for potential bioleaching are mesophilic, growing optimally at temperatures between 30 and 45 °C. Shaking or stirring is essential to ensure optimal growth conditions and prevent biomass coalescence.

**Figure 1:**
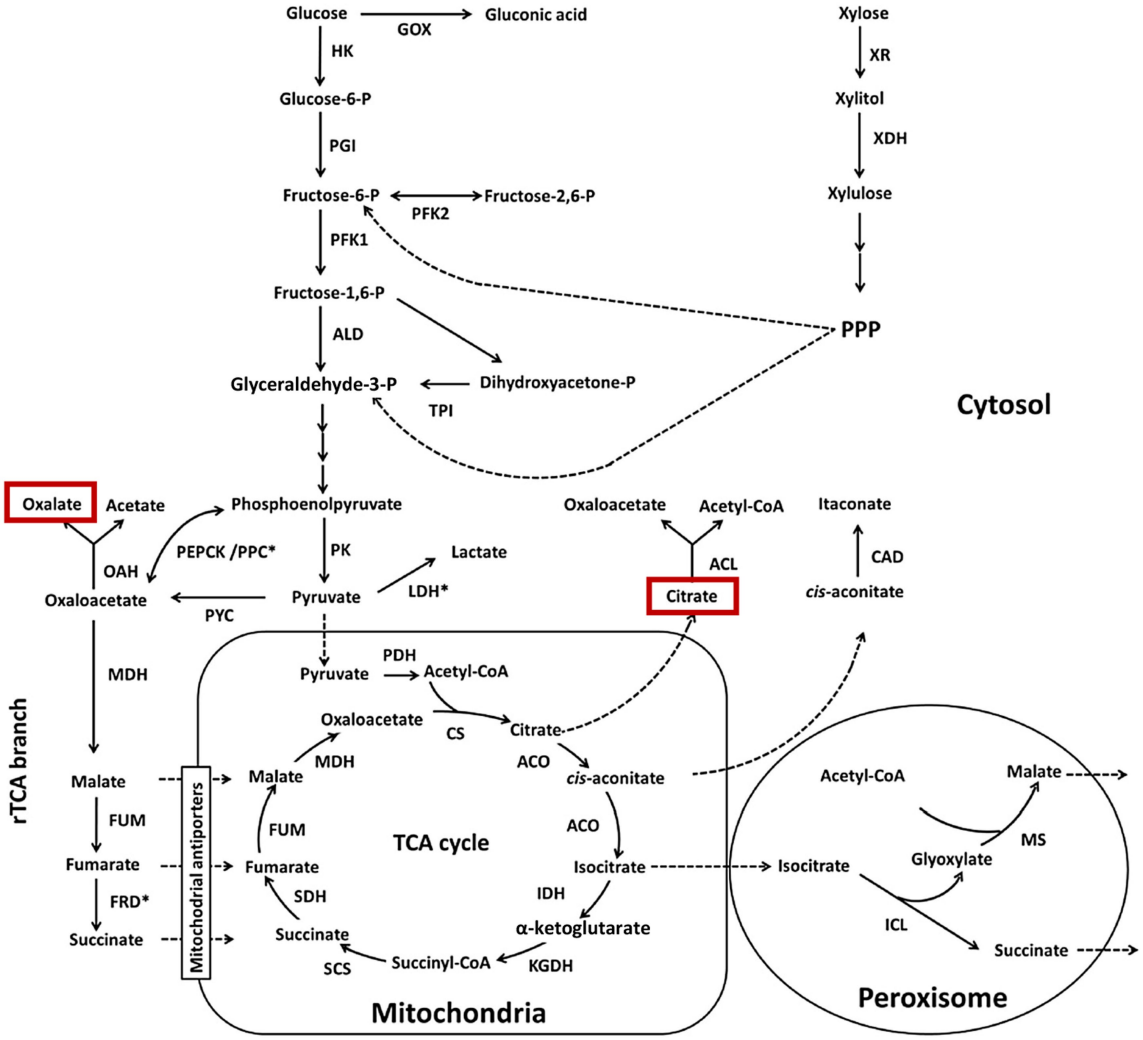
Citric acid and oxalic acid formation in fungal metabolism. For background information on metabolism and enzyme abbreviations, the reader is referred to the original source. Yang et al. (2017), with permission from Elsevier Co.

Citric acid formation (Figure 1) is a well-known pathway in microbes, and the production of oxalic acid is also well documented in bacteria and fungi (Berovic and Legisa, 2007; Show et al., 2015; Behera et al., 2021; Yang et al., 2017; Palmieri et al., 2019). Fungi typically produce citric and oxalic acids in higher proportions than other organic acids, and their concentrations directly impact the pH of culture solutions as well as uranium dissolution. The sources of carbon and the time course of fungal cultures affect the concentrations and relative mixtures of acid metabolites. The action of organic acids on uranium minerals is attributed to the formation of soluble hexavalent uranyl-carboxylate complexes (Wang et al., 2024). Optimizing and controlling culture conditions is important because fungi primarily use carbon sources for biomass growth, leading to less carbon being metabolized into organic acids. Continuous culture approaches, such as fixed-film systems that maintain high biomass and avoid batch growth limitations, appear promising in redirecting the flow of carbon from biomass requirements to organic acid production. However, this approach has yet to be tested in uranium leaching experiments.

The leaching reactions are most effective at a pH of 1.5 to 3.5, as the low pH promotes proton attack on the minerals and reduces uranium precipitation in the leach solution. Acidolysis serves as the primary mechanism through which protons and organic acids function as lixiviants for uranium solubilization. Organic acids form coordinate-covalent bonds via carboxyl (COOH^−^), hydroxyl (OH^−^), or other functional groups with dissolved uranyl ions (Schmeide and Bernhard, 2008; Dodge and Francis, 2003; Bhatti and Yasmin, 2001; Li et al., 1980). The creation of metal-complexing ligands, such as organic acids produced by fungi, enhances the dissolution and solubility of not only uranium but also many other metals and metalloids (Ghazala et al., 2019; Harpy, 2021; Amin et al., 2014; Abhilash and Pandey, 2013; Patra et al., 2011; Mishra et al., 2009; Hefnawy et al., 2002).

X-ray absorption near-edge spectroscopy (XANES) spectra indicated uranyl coordination with carboxylate ligands, suggesting that carboxylic acids are involved in uranium dissolution (Fomina et al., 2008). Many organic acids, including short-chain fatty acids, form soluble complexes with uranium. Various organo-uranium compounds have been reported in the literature, including uranyl formate (UO_2_(HCOO)_2_·H_2_O), U(IV)-formate (U(HCOO)_4_), uranyl acetate (UO_2_(CH_3_COO)_2_·2H_2_O), U(IV)-acetate (U(CH_3_COO)_4_), alkali-uranyl acetate (Na/KUO_2_(CH_3_COO)_3_), uranyl oxalate (UO_2_C_2_O_4_·3H_2_O), and U(IV)-oxalate (U(C_2_O_4_)_2_·6H_2_O). Uroxite and metauroxite are known oxalate complexes of uranium mineralization (Kampf et al., 2020). Uranium-bearing thucholite exhibits a variable composition of complex polymerized organic matter (Hoekstra and Fuchs, 1960; Luo et al., 2014; Khan et al., 2020; Syczewski et al., 2024).

Citric acid and oxalic acid are the most effective leaching agents for uranium dissolution from silicate, oxide, and sedimentary-type uranium-containing ores. The attack of citric acid and oxalic acid on uranium minerals releases UO_2_^2+^ in the leach solution through acidolysis and sequesters uranium as soluble UO_2_^2+^-citrate and UO_2_^2+^-oxalate complexes. UO_2_^2+^ forms complexes with all anionic forms of citric acid. In an aqueous solution, citric acid and oxalic acid are fully dissociated into citrate (C_6_H_5_O_7_^3−^), oxalate (C_2_O_4_^2−^), and H^+^ ions. Citric acid is a hydroxy-tricarboxylic acid and contains three carboxyl groups (pKa_1_ = 3.13; pKa_2_ = 4.76; pKa_3_ = 6.39) and one hydroxyl group (pKa_4_ = 10.82). Oxalic acid (C_2_H_2_O_4_) has two carboxyl groups (pKa_1_ = 1.20 and pKa_2_ = 4.20 at 25 °C) that can donate protons (Skoog et al., 1992; Sillen and Martell, 1964). Under oxidizing conditions, citrate ions (C_6_H_5_O_7_^3−^) form bi-dentate complexes (UO_2_-citrate)_2_^−^ with uranyl ions and, to a lesser extent, tridentate complexes (UO_2_-citrate)^−^ (Martell and Smith, 1977). The binary dimeric complex (UO_2_)_2_(HCit-H)_2_^−^ is well established and exists in the pH range of 2.0–7.0 (Hummel et al., 2005). Speciation changes due to the polymerization of the monomer [UO_2_(cit)]^−^ begin at approximately pH 1.0, forming a dimer [(UO_2_)_2_(cit)_2_]^2−^ at a pH of 1.8 and above. The dimer [(UO_2_)_2_(cit)_2_]^2−^ is bridged via hydroxyl groups of the citrate ligand. Ohyoshi et al. (1975) reported the formation of three types of uranyl citrate complexes: UO_2_H_3_Cit^+^, UO_2_H_2_Cit, and UO_2_H_3_Cit^−^ at a pH range of 2.2–2.9. At pH 2.28, the dominant complex is UO_2_H_3_Cit^+^. The uranyl-citrate dimeric complex (UO_2_)_2_(HCit_−H_)_2_^−^ is formed in acidic aqueous solutions. At higher pH values, ternary dimeric mono- and bis-hydroxo and trimeric complexes are formed: (UO_2_)_2_(HCit_−H_)_2_(OH)^3−^, (UO_2_)_2_(HCit_−H_)_2_(OH)_2_^4−^, (UO_2_)_3_(O)(Cit_−H_)_3_^8−^, and (UO_2_)_3_(O)(OH)(Cit_−H_)_2_^5−^ (Kretzschmar et al., 2021). Uranyl ion complexes with several carboxylic acids in the leaching solution are summarized in Table 2. Other low-molecular-weight carboxylic acids produced in substantial concentrations by filamentous fungi include acetic (C_2_H_4_O_2_), malic (C_4_H_6_O_5_), succinic (C_4_H_6_O_4_), and lactic (C_3_H_6_O_3_) acids, which can also dissolve and sequester uranium as carboxylate complexes. Citric acid is of primary interest, and the genetics and biochemistry of the pathway in *Aspergillus* spp. have been thoroughly characterized.

**Table 2:** Examples of soluble uranyl complexes (Lucks et al., 2012; Kretzschmar et al., 2021).

Reaction	Equation
Dissociation of citric acid	C_6_H_8_O_7_ + H_2_O ⇄ C_6_H_7_O_7_^−^ + H_3_O^+^
	C_6_H_7_O_7_^−^ + H_2_O ⇄ C_6_H_6_O_7_^2−^ + H_3_O^+^
	C_6_H_6_O_7_^2−^ + H_2_O ⇄ C_6_H_5_O_7_^3−^ + H_3_O^+^
Complexation/chelation of citric acid	2UO_2_^2+^ + 2Cit^3−^ ⇄ Cit(UO_2_)_2_(HCit_-H_)_2_^2−^
	2UO_2_^2+^ + 2Cit^3−^ + H_2_O ⇄H^+^ + Cit(UO_2_)_2_(HCit_-H_)_2_(OH)^3−^
	2UO_2_^2+^ + 2Cit^3−^ + 2H_2_O ⇄2H^+^ + Cit(UO_2_)_2_(HCit_-H_)_2_(OH)_2_^4−^
	3UO_2_^2+^ + 3Cit^3−^ + H_2_O ⇄5H^+^ + (UO_2_)_3_(O)(Cit_-H_)_3_^8−^
	3UO_2_^2+^ + 2Cit^3−^ + 2H_2_O ⇄5H^+^ + (UO_2_)_3_(O)(OH)(Cit_-H_)_2_^5−^
	3UO_2_^2+^ + 2C_6_H_5_O_7_^3−^ ⇄ (UO_2_)_3_(C_6_H_5_O_7_)_2_
Dissociation of oxalic acid	C_2_H_2_O_4_ + H_2_O ⇄ C_2_HO_4_^−^ + H_3_O^+^
	C_2_HO_4_^−^ + H_2_O ⇄ C_2_O_4_^2−^ + 2H_3_O^+^
Complexation of oxalic acid	UO_2_^2+^ + C_2_O_4_^2−^ ⇄ UO_2_C_2_O_4_
Dissociation of acetic acid	CH_3_CO_2_H + H_2_O ⇄ CH_3_CO_2_^−^ + H_3_O^+^
Complexation of acetic acid	UO_2_^2+^ + CH_3_CO_2_^−^ ⇄ UO_2_CH_3_CO_2_^+^
	UO_2_^2+^ + 2CH_3_CO_2_^−^ ⇄ UO_2_(CH_3_CO_2_)_2_
	UO_2_^2+^ + 3CH_3_CO_2_^−^ ⇄ UO_2_(CH_3_CO_2_)_3_^−^
Dissociation of succinic acid	(CH_2_)_2_(CO_2_H)_2_ + H_2_O ⇄(CH_2_)_2_(CO_2_H)(CO_2_)^−^ + H_3_O^+^
	(CH_2_)_2_(CO_2_H)(CO_2_)^−^ + H_2_O ⇄ (CH_2_)_2_(CO_2_)_2_^2−^ + H_3_O^+^
Complexation of succinic acid	UO_2_^2+^ + (CH_2_)_2_(CO_2_H)(CO_2_)^−^ ⇄ UO_2_(CH_2_)_2_(CO_2_H)(CO_2_)^+^
	UO_2_^2+^ + (CH_2_)_2_(CO_2_)_2_^2−^ ⇄ UO_2_(CH_2_)_2_(CO_2_)_2_
	UO_2_^2+^ + 2(CH_2_)_2_(CO_2_H)(CO_2_)^−^ ⇄ UO_2_(CH_2_)_2_(CO_2_H)(CO_2_)_2_^−^
Dissociation of malonic acid	CH_2_(CO_2_H)_2_ + H_2_O ⇄CH_2_(CO_2_H)(CO_2_)^−^ + H_3_O^+^
	CH_2_(CO_2_H)(CO_2_)^−^ + H_2_O ⇄ CH_2_(CO_2_)_2_^2−^ + H_3_O^+^
Complexation of malonic acid	UO_2_^2+^ + CH_2_(CO_2_H)(CO_2_)^−^ ⇄UO_2_CH_2_(CO_2_H)(CO_2_)^+^
	UO_2_^2+^ + CH_2_(CO_2_)_2_^2−^ ⇄ UO_2_CH_2_(CO_2_)_2_
	UO_2_^2+^ + 2CH_2_(CO_2_H)(CO_2_)^−^ ⇄UO_2_CH_2_(CO_2_H)(CO_2_)_2_^−^

Organometallic complexes on mineral surfaces polarize bonds within the mineral lattice, weakening or cleaving molecular orbital bonds, which leads to the further dissolution of metals (Jones et al., 2003; Zhang and Bloom, 1999). The proposed chemical structure of the binuclear complex, comprising two uranyl ions and two citric acid molecules with four carboxylic groups and two hydroxyl groups, varies with solution pH (Pasilis and Pemberton, 2003), the ratio of uranium to citrate (Rajan and Martell, 1965), temperature (Bailey et al., 2005), and the presence of other soluble metal ions (Dodge and Francis, 2003). Soluble organo-uranium complexes (e.g., uranium-citrate complexes) and insoluble organo-uranium complexes (e.g., uranium-oxalate complexes) can coexist under acidic leaching conditions. Oxalic acid exhibits a higher degree of hydrogen liberation and a stronger chemical affinity than other organic acids, and both attributes facilitate uranium leaching (Kareem et al., 2010).

The leaching of uranium using fungal enrichment cultures or isolates involves either a one-step or two-step leaching process. In the one-step leaching process, the mycelia are in liquid culture and in contact with suspended finely ground ore. This contact can begin immediately after culture inoculation; however, more commonly, the ore material is suspended in a fully grown culture that contains carboxylic acids resulting from fungal fermentation. The culture is typically grown with glucose or sucrose as a carbon source, which leads to the production of carboxylic acids through fermentation. In the two-step leaching process, after the fungal liquid culture has fully grown and produced carboxylic acids, the biomass is separated, usually by filtration, and the ore material is suspended in the spent culture solution. The biomass can then be recycled for the next batch of fermentation. In this method, the contact of spent culture filtrate with the ore prevents uranium sorption in the biomass, unlike the one-step leaching process. Recovering the absorbed uranium from the biomass in the one-step process requires a separate desorption cycle, making it impossible to recycle the biomass for another batch of carboxylic acid production. The production of carboxylic acids can be streamlined in continuous fixed film systems, which do not necessitate biomass separation due to the immobilization of biomass. Citric acid serves as a feedstock in various industrial sectors—such as food, pharmaceuticals, biopolymers, cosmetics, and chemicals—and its industrial production through fermentation is well-established by major biotechnology companies worldwide (Max et al., 2010; Książek, 2024), potentially serving as a model for designing large-scale production capabilities for carboxylic acids in leaching processes.

**Figure 2:**
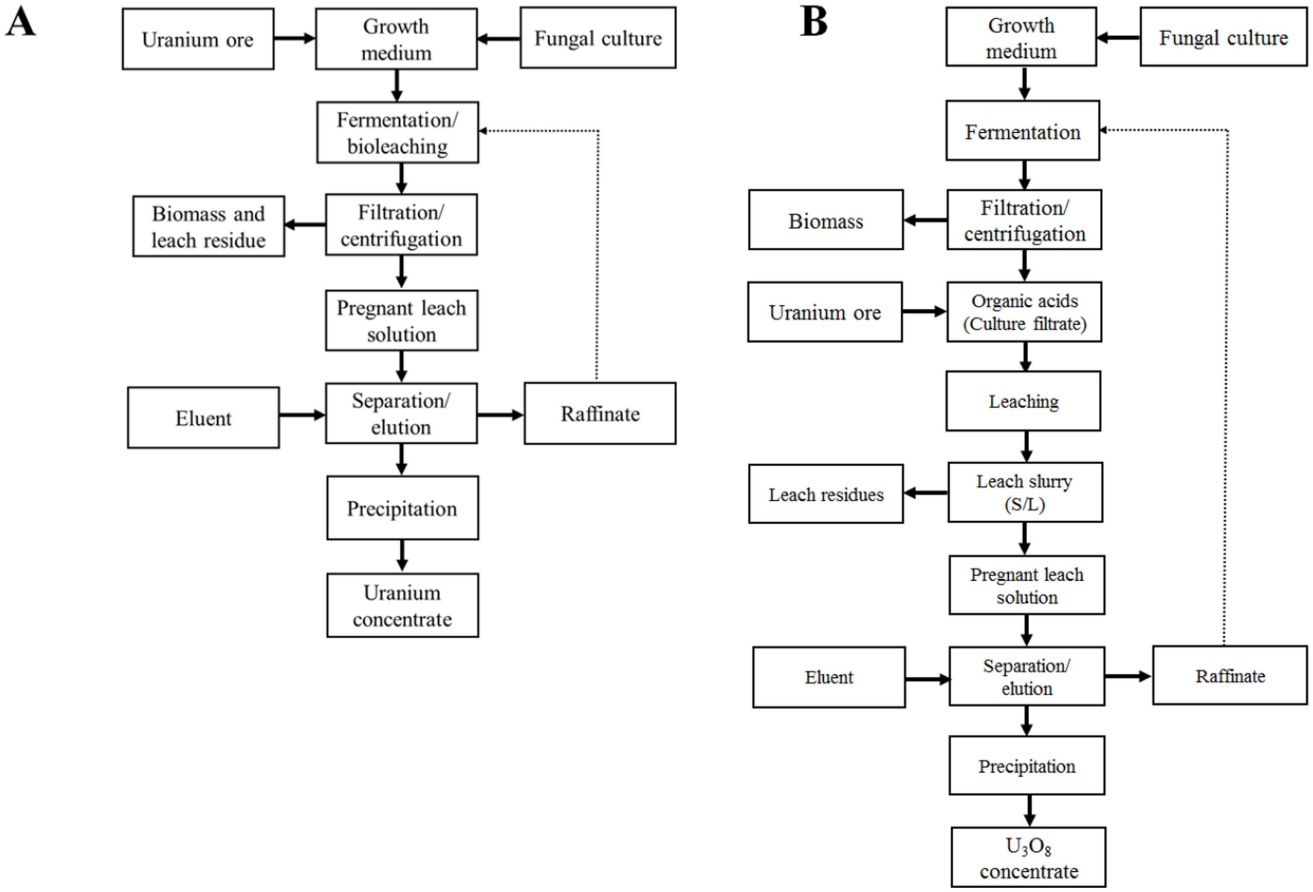
Flowsheets of uranium bioleaching by filamentous fungi **(A)** one-step process and by their carboxylic acid metabolites **(B)** two-step process. One-step and two-step processes are also termed the direct and indirect fungal bioleaching in the literature.

Uranium bioleaching using direct biomass contact (one-step process) and spent medium contact (two-step process) is illustrated in flowsheets in Figure 2. Growth conditions, temperature, contact time, pulp density, uranium mineralogy, and the concentration and composition of organic acids are important parameters for the leaching process. Many minerals contain components that react with organic acids, increasing the acid demand and causing the dissolution of other constituents in addition to uranium. Very little systematic testing of parameters affecting the fungal leaching of uranium has been published (e.g., Mohamed et al., 2024; Varnava and Pashalidis, 2024). The available test results are ore-specific, raising questions about the applicability of these parameter test results to other types of uranium mineralization. Nonetheless, the range and trends of test parameters remain relevant.

## 3 Case studies on the one-step leaching of uranium (direct process)

Mishra et al. (2009) isolated *Cladosporium oxysporum*, *Aspergillus flavus*, and *Curvularia clavata* from water samples collected in the Jaduguda, Bhatin, and Nawapahar uranium mines located in Jharkhand State, India. The fungal cultures were tested for their ability to leach uranium from a low-grade oxide ore sample obtained from the Turamdih uranium mine. This ore sample contained quartz (SiO_2_), alumina (Al_2_O_3_), and magnetite (Fe^II^Fe₂^III^O₄) as major phases, with Al-silicate, Fe-silicate, and hematite (Fe_2_O_3_) present as minor phases. Uraninite (UO_2_) was the main U-mineral, disseminated within schistose rocks. All cultures solubilized between 50 and 71% of U with a 10-day contact time at 30 °C from the Turamdih ore sample (10% pulp density, 0.03% U_3_O_8_ = 0.025% U).

Wang et al. (2012) compared two media for culturing *A. niger*, followed by the leaching of uranium ore. The results showed that the concentration and composition of organic acids were affected by media, pH, and temperature, with pH being the most important factor in uranium leaching. Shen et al. (2023) demonstrated the effect of the carbon source on the bioleaching of rare earth elements through organic acids from *A. niger,* as the concentrations and ratios of citric acid and oxalic acid varied with medium composition. Youssef (2007) reported that *A. niger* achieved 82% U and 76% Cu dissolution from a gibbsite (Al(OH)_3_) mineralization sample containing 0.12% U and 0.7% Cu. The sample was collected from a mineralization site in West Central Sinai.

Kamal et al. (2012) tested *A. niger* and *Penicillium* sp. for the leaching of four Egyptian phosphatic rock samples from the El Sebaiya, Safaga, and Abu Tartur localities. The samples contained 0.003–0.007% uranium, 16.6–26.9% P_2_O_5_, and 0.017–0.137% rare earth elements. Uranium leaching reached 24–28% after 6 days of contact with *A. niger* cultures, along with the dissolution of 31–33% phosphate and 16–18% rare earth elements. The *Penicillium* culture achieved comparable yields of uranium dissolution. The fungi were grown with sucrose and produced citric and oxalic acids, which form poorly soluble complexes with rare earth elements, thereby affecting their recovery in the leaching experiments. Saaed et al. (2002) and Elbarbary et al. (2017) also reported that organic acids in *A. niger* cultures could be used to dissolve uranium from samples of Jordanian fluorapatite and the Abu Tartur phosphate ore, respectively.

Hassanien et al. (2014) reported a solubilization rate of 64–75% for rare earth elements from a (Th-U)-concentrate and a monazite sample during batch direct mode leaching experiments, which contained 2.12 and 0.35% U, respectively. The yields of rare earth elements with *Aspergillus ficuum* and their time course were affected by multiple parameters, including pulp density, pH, carbon source, dominant acids and their concentrations, temperature, and shaking speed.

In uranium leaching studies conducted by Amin et al. (2013, 2014), *A. niger* and *A. terreus* exhibited efficiencies of 34–57% in uranium bioleaching from three samples of uraniferous sedimentary rocks (sample 1: sandy dolostone, dolomite, and gypsum, 0.08% U; sample 2: grey shale with sulfur patches, kaolinite, and quartz, 0.066% U; sample 3: sandy dolostone with a shale interlayer, dolomite, ferron dolomite, quartz, 0.0077% U), sourced from Southwestern Sinai. The fungi produced mixtures of organic acids (acetic, ascorbic, citric, formic, malonic, malic, and oxalic acids) during the bioleaching process, using a 3% pulp density, a 7-day contact time and a temperature of 30 °C. Six other isolates (*A. fumigatus*, *A. flavus*, a *Mucor* sp., and three *Penicillium* spp.) were also tested from the ore samples but demonstrated lower efficiency compared to *A. niger* and *A. terreus*, possibly due to differences in organic acid production.

Hefnawy et al. (2002) isolated seven filamentous fungi from two uranium ore deposits in the Alloga region (West Central Sinai): *A. flavus*, *A. terreus*, *Penicillium brevicompactum*, *P*. *oxalicum*, *P. purpurescens*, *P. lividum*, and *P. spinulosum*. The isolates were tested for uranium bioleaching from two samples (0.4 and 0.25% U) of ferruginous siltstone ores. *A. terreus* and *P. spinulosum* exhibited relatively high uranium leaching efficiencies, suggesting optimal organic acid production compared to the other five isolates. The maximum uranium dissolution of 73–81% from the two ore samples (−200 mesh = −74 μm) was achieved at a 1% pulp density at 28 °C over 7 days. Increasing pulp densities reduced the relative yields of uranium leaching. Fungal mycelium in these experiments sequestered 9.6–17% of uranium from the ore sample, depending on the species, ore sample, and pulp density conditions. Biomass sorption of uranium from solution and its disposal not only results in a loss of uranium recovery but also may complicate compliance with waste regulations.

Ibrahim et al. (2013) tested isolates of *A. niger*, *A. flavus*, *P. lividum*, *P. spinulosum*, and *P*. *oxalicum* from the Nubia sandstone ores of Wadi Natash in the Eastern Desert of Egypt for the bioleaching of uranium and yttrium from two sandstone ore samples (particle size distribution −200 μm). The ore samples contained 0.0065% U and 0.1869% Y (sample 1), and 0.015% U and 0.167% Y (sample 2). *A. niger* showed the highest leaching efficiencies, solubilizing 66 and 61% U, as well as 34 and 51% Y from the two ore samples, respectively, in 7 days at 30 °C, while *A. flavus* solubilized 60 and 28% U, and 58 and 36% Y. These data suggest differences in the concentrations of organic acids in the fungal fermentation media.

*A. sulphureus*, isolated from the El-Sella Desert in Egypt, processed ore material (0.117% U) and leached 83% U over 9 days of incubation at 0.5% pulp density and 30 °C in shake flasks (Hussien et al., 2021). Glucose and ammonium chloride were the optimal C and N sources for the culture. Uranium solubilization was attributed to 45 mg/L of gallic acid (C₆H₂(OH)₃CO₂H, a trihydroxybenzoic acid) and 17 mg/L of ellagic acid (C_14_H_6_O_8_, a heterotetracyclic acid) produced by *A. sulphureus*. Uranium was sequestered as soluble uranylgallate and uranylellagate complexes. Similarly, Elsayad et al. (2020) reported uranium dissolution from the El-Sella ore samples in culture filtrates of *Epicoccum nigrum* that contained gallic and ellagic acids. Gallic acid is an intermediate in the shikimate pathway, linking carbohydrate utilization to aromatic metabolites (Werner et al., 1997). Ellagic acid can be produced by many filamentous fungi, including *Aspergillus* spp., from tannin as the parent polymer material (Sepúlveda et al., 2011). Gallic acid is known to form complexes with uranium, and some studies suggest that it may enhance uranium excretion from body mass (Ortega et al., 1989; Domingo et al., 1990).

Abdelsalam et al. (2021) reported that uranium leaching rates of 55–57% were observed in two *A. nidulans* cultures (strains FII-6 and FII-5) derived from an ore sample containing 0.1872% U. The cultures were tested in shake flasks at a pulp density of 1% and maintained at a temperature of 30 °C with a contact time of 24 h. Leaching in cell-free spent media reached 84% from the same uranium ore sample under comparable conditions (Abdelsalam et al., 2021).

*Aspergillus lentulus*, *A. flavus*, *A. niger*, *A. felis*, and *A. fumigatus,* isolated from a uraniferous black shale of the Um-Bogma Formation in Allouga, Sinai, Egypt, were tested for uranium bioleaching from the black shale (0.165% U) and tailings residue (0.0302% U) in shake flasks (Harpy et al., 2022). The black shale sample contained coffinite [U(SiO₄)_1 − x_(OH)₄ₓ], brannerite [UTi_2_O_6_], autunite [Ca(UO_2_)_2_(PO_4_)_2_·10-12H_2_O], and uranophane [Ca(UO_2_)_2_(SiO_3_OH)_2_·5H_2_O] as uranium minerals. *A. lentulus*, which produced acetic, ascorbic, citric, and oxalic acids, showed the highest leaching efficiency of 73% U from the black shale sample using 3% pulp density at 30 °C under shaking conditions for 7 days (Harpy et al., 2022). *A. lentulus* solubilized 82% U from a tailings residue sample during 8 days of contact time under similar conditions.

Sun et al. (2020, 2022) reported that uranium ore formed pellets (termed bio-ore pellets) with mycelia in *A. niger* cultures. Initially, mycelia and ore particles (−106 μm) created flaky pellets measuring 1–2 mm in diameter, which continued to accumulate into larger clusters of up to 5 mm, stabilized by electrostatic interactions, organic acids, and adhesion to mycelia. During the stable phase, organic acids accumulated, and uranium leaching reached 80% (Sun et al., 2022). Ore particles within the pellets exhibited signs of erosion, primarily attributed to organic acid attacks.

Li et al. (2022) investigated the solubilization of uranium with *A. niger* in the presence of macroparticles (40 g/L, Ø 0.5 mm glass beads) during shake flask experiments using spent culture filtrates. Yields of 75% uranium extraction were achieved from a sample of carbonaceous-siliceous-pelitic uranium ore (−140 mesh = −105 μm, 0.045% U) with mechanical stress provided by shaking at 180 rpm with glass beads. In the absence of glass beads, uranium leaching was approximately 69% U under otherwise similar conditions. Oxalic and citric acids were the primary organic acids involved in this process (Li et al., 2022). The specific reasons for the positive effect of glass beads on uranium leaching remain unclear. Generally, non-substrate particles in fungal cultures have positive effects on mass transfer, growth yields, and metabolic stabilization (Karahalil et al., 2019). Properties of particles such as surface charge, shape, density, and hydrophobicity can also impact mycelial pellet morphology and mycelia-particle clusters.

**Figure 3:**
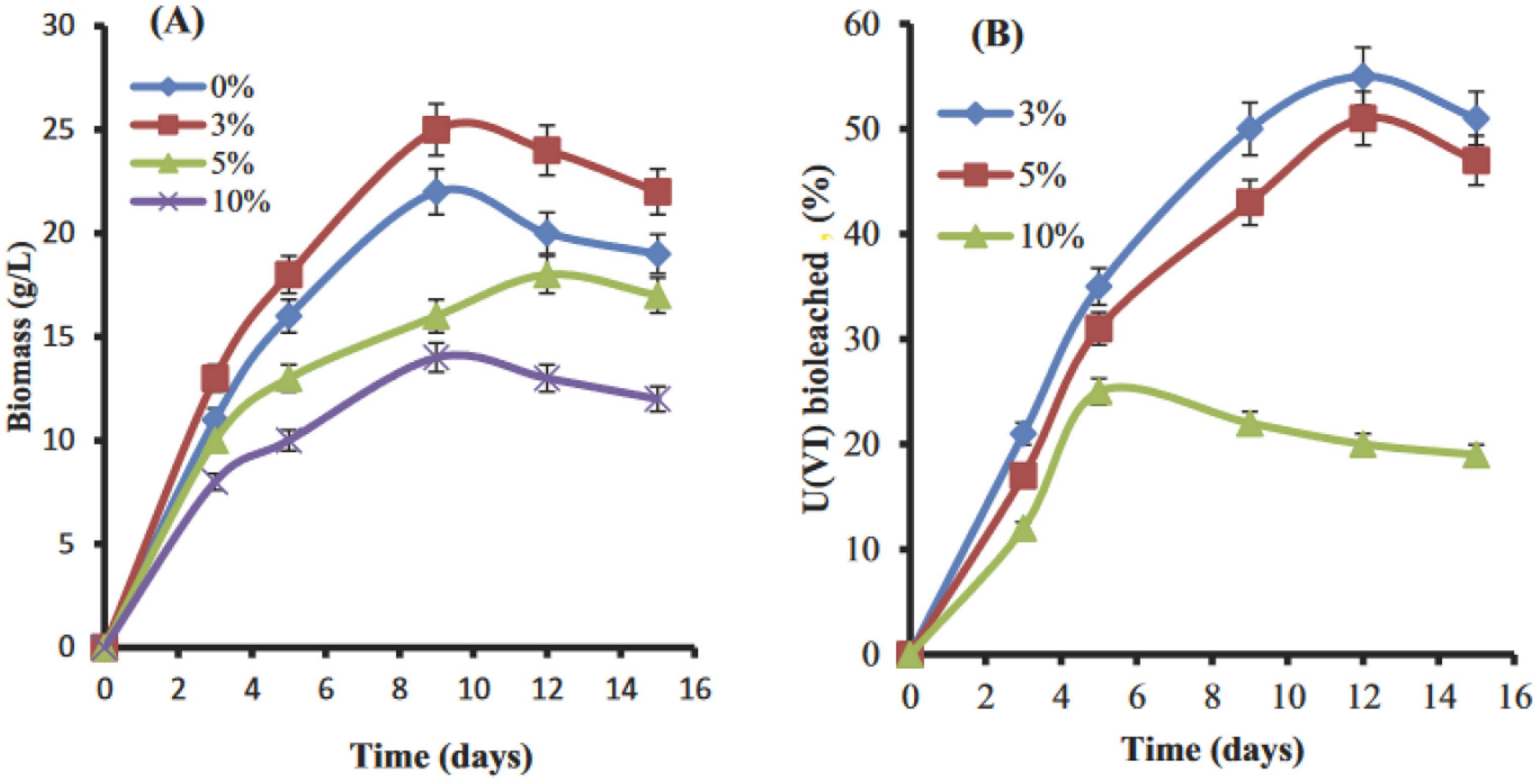
One-step leaching of uranium from a granitic rock sample in shake flasks at 3, 5, and 10% pulp densities at 28-30 °C. **(A)** Growth expressed as dry weight biomass of *A. clavatus*; **(B)** the associated increase of dissolved uranium in the solution phase. Hussien (2020), with permission from Francis and Taylor Co.

Hussien et al. (2016) reported on cultures of *Penicillium purpurogenium* that yielded 72 and 56% bioleaching of uranium from an ore sample (0.035% U_3_O_8_ = 0.03% U) and a waste rock sample (0.0202% U_3_O_8_ = 0.017% U) at a 10% pulp density after 9 days at 30 °C in shake flasks. Subsequently, Hussien (2020) reported on uranium leaching by *A. clavatus* from samples of the El-Sella mineralization, which contained 0.117% uranium distributed in uranophane [Ca(UO_2_)_2_(SiO_3_OH)_2_·5H_2_O], dimorph uranophane-*β*, and autunite [Ca(UO₂)₂(PO₄)₂·10-12H₂O]. The host rock consisted of potash-feldspar, plagioclase, muscovite, and biotite. The yields of uranium leaching ranged from 55 to 67% with a 3% pulp density (−60 mesh = −250 μm) at 28-30 °C over 13 days (Figure 3). The lowest extent of uranium leaching occurred with a 10% pulp density. This may be attributed to the inhibition of mycelial growth by the uranium ore particles at 10% pulp density, as well as biomass sorption of an unknown fraction of dissolved uranium during the one-step bioleaching process. In the presence of biomass, uranium is also re-precipitated as Ca, U-phosphate on the mycelia (Hussien, 2020). Additional examples of one-step bioleaching processes are listed in Supplementary Table S1.

Fungal cell walls contain phosphoryl and carboxyl functional groups, which possess a negative charge and can sequester UO_2_^2+^ (Tobin et al., 1990; Zhang et al., 2020; Lei et al., 2023). Uranyl ions form poorly soluble phosphate complexes that precipitate on fungal hyphae, as shown by Liang et al. (2015) using an *A. niger* culture grown in the presence of uranyl nitrate (UO_2_(NO_3_)_2_) and glycerol 2-phosphate. Fomina et al. (2007) demonstrated that several filamentous fungi could dissolve uranium trioxide (UO_3_) and triuranium octaoxide (U_3_O_8_), resulting in the formation of uranyl-phosphate complexes that accumulated as precipitates on hyphae. The results also suggested the formation of uranyl-carbonate complexes, including secondary U-minerals (Gadd, 2017). Fungus-mediated phosphate complexation can also sequester depleted metallic uranium (Fomina et al., 2008). This biomineralization has been suggested as a bioremediation technique to remove uranium from mine water at uranium mining sites (Schaefer et al., 2021). Uranyl ions are also sequestered in various microbial cell fractions (Ilyas et al., 2022), including extracellular polymers, polyphosphates, DNA, bacterial lipopolysaccharides, peptidoglycan layers, cell wall S-layers, and siderophores. These properties make uranyl acetate a preferred negative stain for biological specimens in electron microscopy.

In addition to uranium precipitating as phosphate and carbonate complexes on fungal mycelia, U(VI) is sequestered in chitin within fungal cell walls (Veglio and Beolchini, 1997; Kapoor and Viraraghavan, 1995; Gadd and Fomina, 2011; Gadd, 2017). Chitin, a polymer (*β*-(1 → 4)-*N*-acetyl-D-glucosamine), is typically the second most abundant polysaccharide in the primary structure of the fungal cell wall, where it is linked to proteins and phosphorylated glucan (Rinaudo, 2006; Brauer et al., 2023; Liu et al., 2023). Mycelial contact involves uranium bonding with the nitrogen of the *N*-glucosamine in chitin, followed by the nucleation and hydrolysis of uranium hydroxide within the chitin network, as detailed by Tsezos (2014).

Filamentous fungi typically produce hydroxamate and carboxylate types of siderophores, with a primary focus on *Aspergillus* species (Renshaw et al., 2002; Pecoraro et al., 2021). The siderophores secreted by fungi effectively bind uranyl ions and other actinides. For example, the siderophore desferrioxamine B (DFO) contains hydroxamate functional groups that form three complexes (UO_2_DFOH_2_, UO_2_DFOH, and UO_2_OHDFOH) with the uranyl ion at pH levels ranging from 3.5 to 10.0 (Mullen et al., 2007). Siderophore sequestration can alter the oxidation states of metals by forming soluble metal-siderophore complexes (metallophores) with divalent and trivalent metal cations, as well as actinides such as Th, U, and Pu (Hofmann et al., 2021; Wang et al., 2022; Schalk et al., 2011). Moreover, siderophores can influence pH conditions, enhancing metal chelation and thereby increasing uranium sequestration (Kalinowski et al., 2004). Osman et al. (2019) purified a trihydroxamate-type siderophore, likely from the ferrichrome structural family, from *A. niger* and tentatively demonstrated its ability to leach uranium and rare earth elements from a low-grade Abu Tartur phosphorite ore sample (0.0023% U, particle size distribution −63 μm). The role of siderophore sequestration in this experiment was unclear because the mass balance of metals relied solely on the solid phase analysis of the ore sample before and after a two-day period. While siderophore deployment may serve a purpose in specialized applications for decontaminating minor spills, this can also be achieved through biomass sorption (Jamir et al., 2024). The large-scale application of siderophores in uranium leaching systems remains uncertain.

Li et al. (2015) established several parameters for U(VI) ion sorption using *A. niger* biomass. Biomass pretreatment and amidoxime (a group of oximes with -NH_2_ as one substituent) enhanced uranium biosorption, which is attributed to uranium binding with -NH_2_ and = NH-OH groups. The sorption of uranium by fungal biomass was developed as an application for treating pregnant leach solutions (Tsezos et al., 1989; Tsezos and McCready, 1991). The test work was piloted with immobilized *Rhizopus* for Denison Mines in the Lake Elliot district; however, the project did not scale up for practical application. Leach solutions in uranium mines also contain other metals, which decrease the specificity and capacity of fungal biomass for uranium retention. Other endeavors reported in the literature focus on developing technology for uranium recovery from leach solutions using microbial biomass (Brierley and Brierley, 1983; Brierley et al., 1986; Tsezos, 2001; Coelho et al., 2020b). The kinetics and mechanisms of biomass biosorption of uranium have been elucidated across numerous fungal genera (Jamir et al., 2024). Furthermore, fungal biomass can be chemically modified to increase its uranium sorption capacity, as reported by Tan et al. (2023). To date, commercial processes for online sequestration of uranium with microbial biomass from bioleaching solutions are not yet operational.

**Figure 4:**
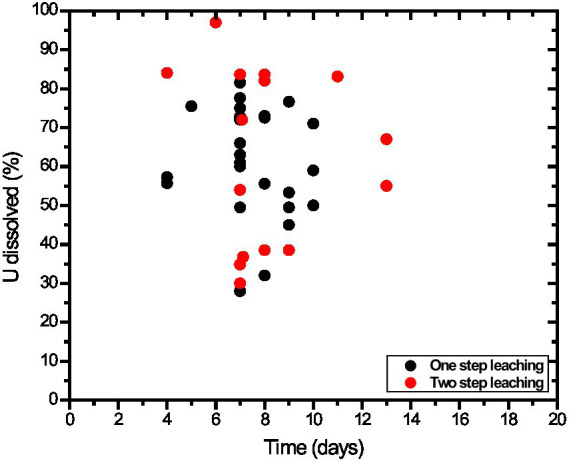
A scatter plot of uranium bioleaching data for filamentous fungi. The scatter plot includes uranium leaching in fungal cultures (one-step process) and culture filtrates (two-step process). The data are for the maximum extent of uranium dissolution in the time course studies presented in the literature. Data are pooled from various literature sources listed in Supplementary Tables S1, S2.

*A. niger* has been the preferred organism for most citric acid-dependent leaching studies, likely due to its well-known use in citric acid production within the industry. There is no systematic comparison of other filamentous fungi and carboxylic acids in uranium bioleaching processes documented in the literature. The yields of uranium leaching by fungi are presented in Figure 4. The data are highly variable, and this variation can be attributed to differences in uranium and host rock mineralogy, pulp density, particle size distribution, pH, carboxylic acid mixtures and concentrations, media formulations, contact time, spent media, temperature, and fungal cultures. Many data sets reported in the literature cannot be summarized on a comparative scale due to the lack of information regarding sample mineralogy, particle size distribution, solution conditions, or carboxylic acid concentrations. Uranium leaching by filamentous fungi has not been systematically investigated with standardized test parameters and protocols, making comparisons among different studies potentially misleading.

## 4 Case studies of uranium leaching using culture filtrates (indirect two-step process)

Uranium leaching by organic acids produced by *A. niger* from sandstone mineralization samples has yielded relatively high results. El-Sheikh et al. (2013) utilized spent culture filtrate from *A. niger* to leach 82% of U from a carbonate-rich latosol rock (0.0875% U and 12.5% CuO, Um-Bogma Formation, SW Sinai, Egypt). The culture filtrate contained formic, citric, acetic, and oxalic acids, with a pH of 2.5–3.0. The sample had a − 200 mesh particle size distribution (−74 μm) and was leached at 50 °C for 24 h. The solution used for Cu leaching contained adipic acid, which increased Cu dissolution to 95%. Adipic acid [(CH_2_)_4_(COOH)_2_] forms soluble copper and uranyl adipates, thereby enhancing leaching yields. For uranium leaching, the leach solution was replaced with a fresh batch of spent culture filtrate that did not contain adipic acid. The rock sample contained carbonate, chloride, sulfate, and phosphate minerals. Although discrete U-mineralization was not specifically identified, uranium was likely associated with the phosphate phase.

*Aspergillus niger* culture filtrates containing organic acids solubilized 83% U_3_O_8_ (equivalent to 71% U) from high-grade uraniferous granite ore (1.134% U_3_O_8_ or 0.962% U) with a contact time of 2 h (Harpy et al., 2022). The sample was ground to −200 mesh (−74 μm) and tested at an ambient temperature of 30 to 39 °C. For reference, the chemical leaching of −60 mesh (−250 μm) sample material using 50 g/L sulfuric acid dissolved 89% uranium at a similar temperature range. Harpy (2021) noted that uranium leaching by culture filtrate results in fewer other metals being dissolved into the lixiviant compared to sulfuric acid leaching, thus providing a higher purity of the final product.

Wang et al. (2012) noted that uranium dissolution from a U-ore sample in *A. niger* spent media ranged between 57 and 82% U over 76 h, although the dominant acids and their concentrations varied with the medium composition. The temperature and pH significantly affected the process. Mixtures of organic acids in *A. ficuum* cultures dissolved 30% uranium and 29% thorium in 24 h at pH 3.0 and room temperature from a U-Th-monazite concentrate (0.75% pulp density, 2.44% U, 19% ThO_2_), which also contained rare earth elements (Desouky et al., 2016). Thorium was selectively precipitated as Th-oxalate using 10% oxalic acid at pH 0.9 prior to the precipitation of uranium as ammonium diuranate with NH_4_OH at pH 5–6. Rare earth elements can be a key economic factor in the bioleaching of uranium ores or tailings (Reynier et al., 2021).

Wang et al. (2015) reported comparable uranium dissolution from pitchblende in a column system using sulfuric acid and a mixture of organic acids in *A. niger* culture filtrates. The time course showed a relatively rapid initial dissolution of uranium within two days, followed by a slower phase. This profile may indicate uranium reactivity on the mineral surface and slow diffusion of the lixiviant into the inner layers. Wang et al. (2015) highlighted the presence of *Aspergillus* spores in the filtrate, which subsequently germinated, leading to undesired mycelial growth in the elution system. Exacerbated biofouling issues may arise even in a low pH environment since a pH of 2 is not prohibitive for some *Aspergillus* spp. Fungal resistance to uranium and other metals is not a limiting factor, as fungal biomass is not in contact with uranium-containing leach solutions in the two-step leaching process.

**Figure 5:**
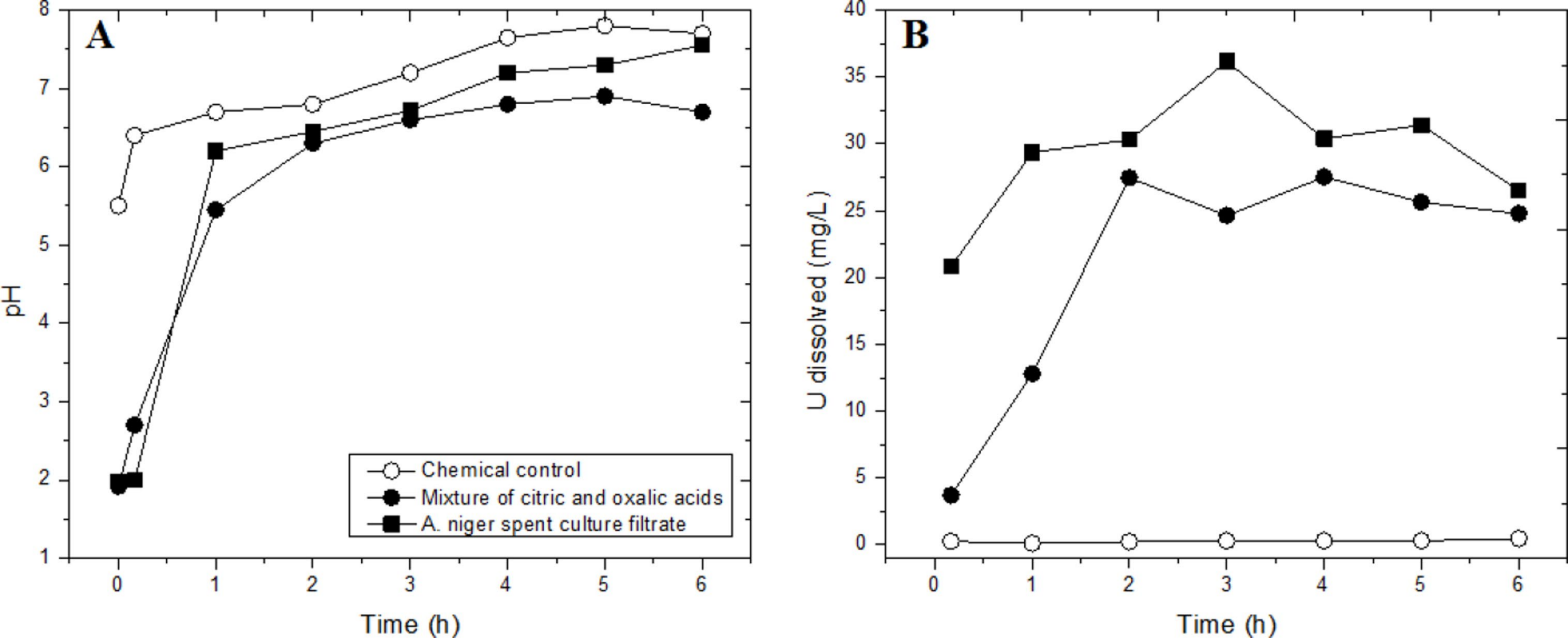
Time course of uranium leaching from a sandstone ore sample (10% pulp density) in a culture of *A. niger* and culture filtrate in shake flasks. **(A)** pH changes; **(B)** dissolved uranium. 100% dissolution of uranium equals 93 mg/L. The chemical control was a batch of sterile medium without inoculation. Adapted from Bhatti and Yasmin (2001).

Spent media of *A. niger* solubilized 97% U and 79% Cu from a Cu-U bearing ore sample (10% pulp density) over a contact period of 6 days at 60 °C (Abd El Wahab et al., 2012). The leaching yield increased when the pH was lowered with additional sulfuric acid. The ore sample was sourced from the upper organic-rich mudstone of the Um-Bogma Formation in the Abu Thor area, West Central Sinai, which contained 0.22% U, 25% CuO, 33% SiO_2_, 10.4% Al_2_O_3_, and 8.5% CaO as major constituents. Bhatti and Yasmin (2001) compared uranium dissolution from a sandstone ore (10% pulp density, −200 mesh = −74 μm, 0.093% U) in spent culture filtrate of *A. niger* and in citric and oxalic acid solutions (Figure 5). The sandstone uranium ore sample was obtained from the Baghalchur mines in the Suleiman Range of Dera Ghazi Khan, Pakistan. The primary uranium mineral in the sandstone ore sample was tyuyamunite [Ca(UO_2_)_2_V_2_O_8_·5-8H_2_O]. Uranium dissolution reached 39% U in culture filtrate and 27% U with the mixed acid solution after a 3 h contact time of 3 h in shake flasks. *A. niger* was cultivated with 55 mM sucrose as the C source for seven days, resulting in the production of 26 mM citric acid and 55 mM oxalic acid. Normalized to the C balance, 8% of the sucrose carbon was metabolized into citric and oxalic acids. The citric and oxalic acid solution was prepared with the same acid concentrations as those in the spent *A. niger* culture filtrate.

Abdelsalam et al. (2021) reported yields of 57 ± 9% U leaching in the test culture and 84 ± 2% U leaching with culture filtrates in 24 h at 30 °C from a − 230 mesh (−63 μm) rock sample (1% pulp density) that contained 0.187% U. The culture was identified as a mixture of *A. niveus* and a *Streptomyces* sp. Additional details of the two-step leaching processes are listed in Supplementary Table S2. Examples of data on uranium leaching in culture filtrates are shown in Figure 4.

Ahmed et al. (2024) investigated several parameters for citric acid production by *A. nidulans*, including C and N sources, pulp densities, pH, and temperature ranges. The filtrates served as leaching solutions with a contact time of 0.5 to 3 h to leach uranium from a finely ground, low-grade uranium ore sample containing 0.0404% U in shake flasks. These experiments optimized the conditions for uranium leaching at relatively low pulp densities (1 to 5 g/L) and may assist in planning for the testing of pilot process parameters. However, the data from the shake flask tests are too preliminary for cost–benefit and life cycle estimations.

## 5 Organic acid chemicals as leaching solutions

By bypassing fungal production, citric and oxalic acids have been tested in chemical leach solutions that contacted uranium ores. For example, citric acid (0.1 M) and oxalic acid (0.1 M) solutions dissolved 97 and 67% of uranium, respectively, in shake flask leaching studies conducted over 20 days with a volcano-litho type ore (0.25% U) containing uraninite (Xu et al., 2019). Citric acid (0.1 M) solubilized 75% of U_3_O_8_ (equivalent to 63.6% U) from a low-grade sandstone ore sample (0.027% U_3_O_8_ = 0.023% U) sourced from Baghalchur (Dera Ghazi Khan, Pakistan) during 10 h of contact time in shake flasks (Bhatti and Malik, 1997). A uranium leaching efficiency of 93% U_3_O_8_ (equivalent to 79% U) was achieved from a carbonaceous shale tailings residue (0.0185% U, solid/liquid ratio 1:1.5) at the Abu Zeneima pilot plant (Southwestern Sinai, Egypt) using a 13% humic acid solution as a lixiviant at 70 °C over 15 days of contact (Khalafalla, 2022). Orabi et al. (2021) tested malonic acid for uranium leaching from uraniferous granite (−75 μm) at 25 °C. The sample was obtained from Gabat El Missikat granite ore in the Central Eastern Desert, Egypt. The dissolution yield of uranium at a solid/liquid ratio of 1/4 exceeded 90% after 5 h of contact with 2 M malonic acid. Uranium dissolution was nearly comparable to that achieved using a 1.5 M sulfuric acid leach solution; however, malonic acid leaching performed slightly better by yielding lower concentrations of other metals leached from the ore. It is unclear why sulfuric acid resulted in somewhat higher yields of other metals compared to malonic acid. Neither the pH values nor any statistical analyses were reported by the authors for these experiments.

Oxalic acid forms uranium complexes (UO_2_(C_2_O_4_)_2_^2−^), which can create solid uranyl oxalate at high concentrations of oxalic acid (Xiong and Wang, 2021). However, thermodynamic diagrams indicate that the solubilities of uranyl oxalate complexes do not lead to precipitation under the ambient temperature and pH conditions relevant to the bioleaching and organic acid leaching of uranium minerals.

Bakry (2021) reported that uranium was leached from a carbonate-rich black shale in southwestern Sinai (0.136% U) using a 60 mM citric acid and calcium citrate (1:1 acid/salt molar ratio) as a lixiviant. The uranium leaching efficiency increased with temperature, rising from 77% at 25 °C to 98% at 65 °C. The process involved a-200 mesh (−74 μm) particle size and a solid/liquid (S/L) ratio of 1/8 over a period of 1.25 h. Uranium was recovered through the addition of isopropyl alcohol, followed by evaporation and precipitation, and then dried to produce sodium diuranate (Bakry, 2021).

Uranium is associated with phosphate deposits (e.g., apatites) in many geographical areas worldwide. Apatite consists of a group of phosphate minerals, typically including hydroxyapatite (Ca_5_(PO_4_)_3_OH), fluorapatite (Ca_5_(PO_4_)_3_F), and chlorapatite (Ca_5_(PO_4_)_3_Cl). Uranium in phosphates can be leached upon contact with organic acids, but other metals and phosphates also dissolve concurrently in fungal cultures (El-Badry et al., 2016; Elbarbary et al., 2017). Citric acid has been used as a leaching solution to extract uranium from rock phosphate (Bashir et al., 2000). Varnava and Pashalidis (2024) tested 1 mM and 10 mM citric acid for the solubilization of uranium from a phosphogypsum sample containing 0.0048% U (48 mg U/kg). Citric acid forms a Ca-citrate complex (Ca_3_(C_6_H_5_O_7_)_2_(H_2_O)_2_) with a limited solubility of approximately 0.95 g/L at ambient temperatures. Increasing the citric acid concentration from 1 mM to 10 mM exceeded the solubility of Ca-citrate, and its precipitation restricted gypsum (CaSO_4_·2H_2_O) dissolution, thereby lowering the leaching rate of uranium.

A selective leaching process was employed to extract uranium and rare earth elements from Abu-Tartur rock phosphate in Egypt using citric acid and calcium citrate without solubilizing the phosphate content (Farag et al., 2015). The rock sample contained 0.003% U and 0.1058% of rare earth elements (Ce, La, Nd, Sm, Y, and Yb). Leaching efficiencies of 100% for U and 84% for rare earth elements was achieved using a 0.5 M citric acid and calcium citrate leach solution (1:1 acid/salt molar ratio) with a solid-to-liquid ratio of 1:3 and a contact time of 0.5 h (Farag et al., 2015).

## 6 Concluding remarks

Progress in bioleaching uranium from rocks and ores through the use of filamentous fungi has been hindered by inconsistent experimental designs and protocols, as well as a lack of uniform, standardized approaches. Some published data on uranium bioleaching by filamentous fungi are fragmented; they often rely on preliminary experiments with low pulp densities and lack comprehensive follow-up testing and optimization. Mixed carboxylic acids effectively leach uranium from carbonate, oxide, and silicate ores and rocks, helping to neutralize acid consumption by gangue minerals. The carbon sources and the duration of fungal cultures affect the concentrations and relative mixtures of acid metabolites. The reasons behind the variations in uranium leaching yields obtained with different carboxylic acids remain unclear due to the diverse experimental conditions across studies, including pH, carboxylic acid mixtures and concentrations, temperature, particle size distribution, uranium mineralogy, and other primary and ancillary minerals. Optimization and controlling culture conditions are crucial for future studies testing filamentous fungi in leaching processes. However, the optimization of process parameters for scaling up to pilot studies has not yet been reported in the literature.

In batch mode, fungi primarily use carbon sources for biomass growth, while less carbon is metabolized into organic acids. Organic waste materials as carbon sources may be suitable for fungal cultivation, but this presents a complex, multifaceted challenge, as it involves other microbes and organic mixtures of varying compositions. Contaminant microbes and recalcitrant solid-phase carbon acting as sorbents of uranium can hinder the bioleaching process. Culture approaches that maintain high biomass, such as continuous flow techniques and chemostats, appear feasible in efforts to divert more of the carbon flow from biomass requirements to organic acid production. Two-stage processes with fungi involve the fermentative production of organic acids, which are then used as lixiviants to dissolve uranium from minerals or ores. Such leaching processes, where filamentous fungi are not in direct contact with minerals, reduce potential biological inhibition caused by the toxicity of mineral phases and dissolved metals. Minimal biomass contact in the leach solution also lowers the fraction of uranium associated with the biomass, thus alleviating the need for uranium elution from the biomass. The cost-effectiveness of the process for piloting and commercialization cannot be assessed at this time because the only data available come from bench-scale experiments of varying sophistication.

Studies on biomass production with predictable organic acid formation have yielded variable results. Data on organic acid consumption during contact with uranium ore have not been presented in the literature despite it being a key parameter in the leaching process. The experiments have been conducted by various research groups. A consortium of these groups could establish unified standards and procedures to advance the level of training, skills, and technology from ambiguity and ordinariness to reproducible and reliable studies on the fungal leaching of uranium ores.

## Glossary

Uranium mineral: Ideal formula
Autunite: Ca(UO_2_)_2_(PO_4_)_2_·10-12H_2_O
Brannerite: UTi_2_O_6_
Coffinite: U(SiO₄)_1 − x_(OH)₄ₓ
Dimorph uranophane-β: Ca(UO_2_)_2_(SiO_3_OH)_2_·5H_2_O
Metauroxcite: (UO_2_)_2_(C_2_O_4_)(OH)_2_(H_2_O)_2_
Thucholite (pyrobitumen): Admixture of uraninite, hydrocarbons, and sulfides
Tyuyamunite: Ca(UO_2_)_2_(VO_4_)_2_·5-8H_2_O
Uraninite: UO_2_
Uranophane: Ca(UO_2_)_2_(SiO_3_OH)_2_·5H_2_O
Uroxcite: [(UO_2_)_2_(C_2_O_4_)(OH)_2_(H_2_O)_2_]·H_2_O
