# Supplementary material for: Bioleaching of uranium from ores and rocks using filamentous fungi
Source: Front Microbiol. 2025 Apr 30;16:1523962. doi: 10.3389/fmicb.2025.1523962 (PMC12075322; doi:10.3389/fmicb.2025.1523962)
Supplement: Supplementary file 1 [file Table_1.docx]

**Supplementary material**

**Table S1:** Bioleaching of uranium from ores in spent fungal cultures, representing one-step bioleaching. The U_3_O_8_ data have been converted to U data. Where possible, the decimals in the leaching data and acid concentrations have been rounded to the closest full digit.

| **Fungal species** | **Uranium deposit** | **Uranium content** | **Lixiviant** | **Experimental conditions** | **Uranium**  **recovery** | **Reference** |
| --- | --- | --- | --- | --- | --- | --- |
| *A. niger* | Uraniferous sedimentary rocks, SW Sinai, Egypt | 0.08% (Ag-1)  0.066% (Ag-2)  0.0077% (Ag-3) | Acetic, citric, formic, and oxalic acids | Shake flasks, 1% PD, 30 °C, 7 d | 43%  54%  64% | Amin et al. (2013) |
| *A. niger* | Carbonaceous-siliceous-pelitic uranium ore, Jiangxi Province, E-China | 0.086% | Oxalic and citric acids | Shake flasks, 4% PD, <140 mesh ± 40 g/L glass beads, 30 °C, 5 d, 35 mM oxalic and 27 mM citric acids without glass beads); 34 mM oxalic and 17 mM citric acid with 0.5 mm glass beads) | 75% (+ beads)  69% (- beads) | Li et al. (2022) |
| *A. niger* | Sandstone G2  Nubia sandstone, Wadi Natash, E-Desert, Egypt | 0.015% | Oxalic and citric acids | Shake flasks, 2% PD, -75 mesh, 35 °C, 7 d, 0.21 mM citric and 0.05 mM oxalic acid | 90% | Ibrahim et al. (2013) |
| *A. sulphureus* | El-Sella, SE-Desert of Egypt | 0.117% | Gallic and ellagic acids | Shake flasks, 2% PD, 28 °C, 175 rpm, 7 d, final pH 4.9, 0.26 mM gallic and 0.06 mM ellagic acid | 50% | Hussien et al. (2021) |
| *A. clavatus* | Granitic rock, Gabal El-Sella, Egypt | 0.117% | Citric, oxalic, and gluconic acids | Shake flasks, two-steps leaching, 3, 5, and 10% PD, 30 °C, 12 d, 61 mM citric, 19 mM gluconic and 6.5 mM oxalic acid | 65% (3% PD)  55% (5% PD)  20% (10% PD) | Hussien (2020) |
| *A. flavis* BM1 | Sandstone ore, Jharkhand mines-UCIL, India | 0.025% | Oxalic, citric, and gluconic acids | Shake flasks, 10% PD, 30 °C, 9 d | 59% | Mishra et al. (2009) |
| *A. nidulans* | Ferruginous sandstone,  Um Bogma Formation in Abu Thor, Sinai, Egypt | 0.045% | Organic acids | Shake flasks, 3% PD, 30 °C, 7 d | 80% | Attia et al. (2022) |
| *A. nidulans* | Uraniferous sedimentary rock, SW-Sinai, Egypt | 0.145% | Organic acids | Shake flasks, 3% PD, 30 °C, 7 d | 83% | Kawady (2021) |
| *A. terreus* | Ferruginous siltstone ores, Egypt | OS1 (0.4%)  OS2 (0.25%) | Oxalic, acetic, citric, formic, malonic, gallic, and ascorbic acids | Shake flasks, 1% PD, 28 °C, 7 d | 75% (OS1)  73% (OS2) | Hefnawy et al. (2002) |
| *A. terreus* | Uraniferous sedimentary rocks, SW-Sinai, Egypt | Ag-1 (0.08%)  Ag-2 (0.066%)  Ag-3 (0.0077%) | Ascorbic, citric, and oxalic acids | Shake flasks, 1% PD, 30 °C, 7 d | 26%  35%  45% | Amin et al. (2013) |
| *A. hollandicus* | Gabal um Hamd, Southwestern Sinai, Egypt; siltstone (W1), gray shale (W2), sandy dolostone (W3) | W1 (0.025%) W2 (0.0391%) W3 (0.1161%) | Oxalic, maleic, quinic, lactic, citric, and butyric acids | Shake flasks, 3% PD, 30-35 °C, 7 d | 74%  64%  78% | Mohamed et al. (2024) |
| *A. niveus* | Rock sample Wadi Naseib, Sinai, Egypt | 0.187% | Organic acids | Shake flasks, 1% PD, -230 mesh, 30 °C, 4 d | 55.7±1.5% | Abdelsalam et al. (2021) |
| *A. nidulans* | Rock sample Wadi Naseib, Sinai, Egypt | 0.187% | Organic acids | Shake flasks, 1% PD, -230 mesh, 30 °C, 4 d | 57.3±5.3% | Abdelsalam et al. (2021) |
| *Cladosporium oxysporum* | Sandstone ore, Jharkhand mines-UCIL, India | 0.025% | Oxalic, citric, and gluconic acids | Shake flasks, 10% PD, 30 °C, 9 d | 71% | Mishra et al. (2009) |
| *Curvularia clavata* | Sandstone ore, Jharkhand mines-UCIL, India | 0.025% | Oxalic, citric, and gluconic acids | Shake flasks, 10% PD, 30 °C, 9 d | 50% | Mishra et al. (2009) |
| *Penicillium spinulosum* | Ferruginous siltstone ores, Alloga region,  W-Central Egypt | OS1 (0.40%)  OS2 (0.25%) | Organic acids | Shake flasks, 1% PD, 28 °C, 7 d | 81% (OS1)  78% (OS2) | Hefnawy et al. (2002) |
| *Penicillium purpurogenium* | Sedimentary ore and waste rock, N-Gabal Gatter, Egypt | 0.032%  0.017% | Citric and oxalic acids | Shake flasks, 10% PD, 30 °C, 9 d, 85 mM citric and 38 mM oxalic acid | 73%  56% | Hussien et al. (2016) |
| *Penicillium citrinum* | Gabal um Hamd, Southwestern Sinai, Egypt; siltstone (W1), gray shale (W2), sandy dolostone (W3) | W1 (0.025%) W2 (0.0391%) W3 (0.1161%) | Oxalic, maleic, quinic, lactic, citric, and butyric acids | Shake flasks, 3% PD, 30-35 °C, 7 d | 74%  64%  78% | Mohamed et al. (2024) |
| *Epicoccum nigrum* | El Sella, SE-Desert, Egypt | 0.1173% | Gallic and ellagic acids | Shake flasks, 0.5% PD, 30 °C, 9 d, 0.7 mM gallic and 0.8 mM ellagic acid | 77% | Elsayad et al. (2020) |

Abbreviation: PD = Ore pulp density.

**Table S2**

Bioleaching of uranium from ores in in spent media of fungal cultures (biomass removed), representing two-step bioleaching. The U_3_O_8_ data have been converted to U data. The decimals in the leaching data have been rounded to the closest full digit.

| **Fungal species** | **Uranium deposit** | **Uranium content** | **Lixiviant** | **Experimental conditions** | **Uranium**  **recovery** | **Reference** |
| --- | --- | --- | --- | --- | --- | --- |
| *A. niger* | Sandstone uranium ore with pitchblende, W-China | 0.10% | Oxalic acid, citric acid, and other organic acids | Column, 10 kg ore, drip at 10.62 L/m^2^·h, -10 mm, 30-37 °C, 15 d, pH 2.3, 26.2 mM oxalic, 4.3 mM citric, 0.07 mM α-ketoglutaric, 3.92 mM acetic, 0.66 mM succinic and 1.26 mM malic acid | 82% | Wang et al. (2015) |
| *A. niger* | Granite uranium ore, China | 0.117% | Citric, oxalic, α-ketoglutaric, and malic acids | Shake flasks, 5% PD, -200 mesh, PSA medium, pH 2.3, 25°C, 1 d, 26.16 mM oxalic, 0.43 mM citric, 0.08 mM α-ketoglutaric, 3.92 mM acetic, 0.66 mM succinic and 1.26 mM malic acid | 82% | Wang et al. (2013) |
| *A. niger* | Granite uranium ore, China | 0.117% | Oxalic and citric acids | Shake flasks, 5 PD%, -75 μm, 25-35 °C, PCS medium, pH 2.0, 1 d, 12.07 mM oxalic, 0.95 mM citric, 0.07 mM α-ketoglutaric and 0.76 mM acetic acid | 68% | Wang  et al. (2012) |
| *A. niger* | Cu-U bearing ore, Um-Bogma Formation,  W-Sinai, Egypt | 0.22% | Organic acids | Shake flasks, 10% PD, 60 °C, 6 d | 97% | Abd El Wahab et al. (2012) |
| *A. niger* | Sandstone, Baghalchur,  Dera Ghazi Khan, Pakistan | 0.093% | Citric and oxalic acids | Shake flasks, 10% PD, <200 mesh, 30 °C, 3 h, 26 mM citric and 55 mM oxalic acid | 37% | Bhatti and Yasmin (2001) |
| *A. niger* | Uraniferous Granite, Gabal Gattar, NE-Desert, Egypt | 0.962% | Organic acids | Shake flasks, 10% PD, -60 mesh, RT,  2 h | 72% | Harpy (2019) |
| *A. ficuum* | Th-U concentrate, Egypt | 2.44% U  19% Th | Oxalic acid | Shake flasks, 0.75% PD, RT, 1 d | 30% U  29% Th | Desouky et al. (2016) |
| *A. lentulus* | Carbonaceous black shale, Um Bogma Formation, Egypt | 0.165% | Acetic, oxalic, ascorbic, and citric acids | Shake flasks, 3% PD, 30 °C, 7 d, 21.60 mM oxalic, 25.0 mM citric, 33.0 mM acetic, and 0.22 mM ascorbic acid | 80% | Harpy et al. (2022) |
| *A. sulphureus* | Granitic rock, El-Sella SE-Desert, Egypt | 0.117% | Gallic and ellagic acids | Shake flasks, 1% PD, 28 °C, 1 d, 150 rpm, final pH 4.82, 0.54 mM gallic and 0.09 mM ellagic acid | 39% | Hussien et al. (2021) |
| *A.* *niveus* | Rock sample, Wadi Naseib, Sinai, Egypt | 0.187% | Organic acids | Shake flasks, 1% PD, -230 mesh, 30 °C, 4 d | 84±2% | Abdelsalam et al. (2021) |
| *A. nidulans* | Rock sample, Wadi Naseib, Sinai, Egypt | 0.187% | Organic acids | Shake flasks, 1% PD, -230 mesh, 30 °C, 4 d | 84±2% | Abdelsalam et al. (2021) |

Abbrevations: PCS = Potato corn syrup; PD = Ore pulp density; PSA = Potato sucrose agar; RT = room temperature.

**References for Tables S1 and S2**

Abdelsalam, S. M., Kamal, N. M., Harpy, N. M., Hewedy, M. A., El‑Aassy, I. E. (2021). Bioleaching studies of uranium in a rock sample from Sinai using some native *Streptomyces* and *Aspergillus* species. *Curr. Microbiol*. 78, 590-603. doi.org/10.1007/s00284-020-02301-y

Abd El Wahab, G. M., Amin, M. M., Aita, S. K. (2012). Bioleaching of uranium-bearing material from Abu Thor area, West Central Sinai, Egypt for recovering uranium. *Arab J. Nucl. Sci. Appl*. 45, 169-178.

Amin, M. M., Elaassy, I. E., El-Feky, M. G., Sallam, A. M., Talaat, M. S., Kawady, N. A. (2013). Bioleaching of uranium by *Aspergillus niger* and *Aspergillus terreus* isolated from uraniferous sedimentary rocks, Southwestern Sinai, Egypt. *Roman. J. Biophys*. 23, 231-247.

Attia, R. M., Sallam, O. R., Abbas, A. E. A., Kawady, N. A. (2022). Comparative evaluation of chemical and bio techniques for uranium leaching from low grade sandstone rock sample, Abu Thor, southwestern Sinai, Egypt. *J. Radioanal. Nucl. Chem*. 331, 5675–5689. doi.org/10.1007/s10967-022-08621-6

Bhatti, T. M., Yasmin, T. (2001). “Bioleaching of uranium from sandstone ore by *Aspergillus niger*,” in *Biohydrometallurgy: Fundamentals, Technology and Sustainable Development: Part B*, eds. V.S.T. Ciminelli, O. Garcia Jr., (Elsevier, Amsterdam), 651-660.

Desouky, O. A., E-Mougith. A. A., Hassanien, W. A., Awadalla, G. S., Hussien, S. S. (2016). Extraction of some strategic elements from thorium–uranium concentrate using bioproducts of *Aspergillus ficuum* and *Pseudomonas aeruginosa*. *Arab. J. Chem*. 9, 795-805. doi.org/10.1016/j.arabjc.2011.08.010

Elsayad, A. M., Hussien, S. S., Mahfouz, M. G., El Mougith, A. A., Hassanien, W. A. (2020). Bioleaching of uranium from El-Sella ore material using *Epicoccum nigrum*. *IOP Conf. Series: Mater. Sci. Eng*. 975:012021. doi:10.1088/1757-899X/975/1/012021

Harpy, N. (2019). Correlation between bio- and classical techniques in uranium leaching of granite sample from Gabal Gattar, North Eastern Desert, Egypt. *Int. J. Environ. Anal. Chem.* 101, 2003-2015. doi:10.1080/03067319.2019.1691187

Harpy N. M., El Dabour, S .E., Nada, A. A., Sallam, A. M., El Feky M. G., El Aassy A. E. (2022). Fungal leaching of uranium from low grade ore and its waste using *Asp*. *lentulus* at Allouga locality, Southwestern Sinai, Egypt. *J. Rad. Nucl. Appl.* 7(3), 41-50. doi.org/10.18576/jrna/07030

Hefnawy, M. A., El-Said, M., Hussein, M., Amin, A. M. (2002). Fungal leaching of uranium from its geological ores in Alloga Area, West Central Sinai, Egypt. *Online J. Biol. Sci*. 2, 346-350. doi.org/10.3923/jbs.2002.346.350

Hussien, S. S., Desouky, O. A., Mohamadey, S. E. (2016). Microbial leaching of uranium from low grade ore and waste sample of northern part of Gabal Gatter, Egypt using *Penicillium purpurogenium* and *Pseudomonas fluorescens* SHA 281. *J. Prog. Res. Biol*. 3, 127-141.

Hussien, S. S. (2020). Microbial leaching of El-Sella mineralisation by *Aspergillus clavatus* – a fact of fungal-uranium interface. *Int. J. Environ. Stud.* 77, 275-296. doi.org/10.1080/00207233.2020.1736441

Hussien, S. S., Mosbah, A. S., El-Mougith, A. A., Hassanien, W. A., Mahfouz, M. G. (2021). Bio-dissolution process as environmental technology for uranium leaching from El-Sella ore material by *Aspergillus sulphureus. Geomicrobiol. J.* 38, 540-548. doi:10.1080/01490451.2021.1883161

Ibrahim, M. E., Abdel Aziz, Z. K., Mira, H. I., Amin, M. M., Morsy, W. M. (2013). Optimization of different physical parameters for bioleaching of uranium and rare earth elements from Nubia sandstones, Wadi Natash, Eastern Desert, Egypt. *Nucl. Sci. Sci. J.* 2, 165-174. doi.org/10.21608/nssj.2013.30992

Kawady, N. A. (2021). Biological solubilization and sorption of uranium from ore sample at Abu Thor area, Southwestern Sinai, Egypt using *Aspergillus nidulans*. *J. Rad. Nucl. Appl*. 7, 27-35. doi.org/10.18576/jrna/07010

[Li](https://www.sciencedirect.com/science/article/abs/pii/S0892687522001030#!), G., [Sun](https://www.sciencedirect.com/science/article/abs/pii/S0892687522001030#!), J., [Li](https://www.sciencedirect.com/science/article/abs/pii/S0892687522001030#!), F., [Wang](https://www.sciencedirect.com/science/article/abs/pii/S0892687522001030#!), Y., [Li](https://www.sciencedirect.com/science/article/abs/pii/S0892687522001030#!), Q. (2022). Macroparticle-enhanced bioleaching of uranium using *Aspergillus niger*. *Miner. Eng.* 180, 107493. doi.org/10.1016/j.mineng.2022.107493

Mishra, A., Pradhan, N., Kar, R. N., Sukla, L. B., Mishra, B. K. (2009). [Microbial recovery of uranium using native fungal strains](http://www.sciencedirect.com/science/article/pii/S0304386X08001205). *Hydrometallurgy* 95, 175-177. doi.org/10.1016/j.hydromet.2008.04.005

Mohamed, W. S., Abbas, Y. M. M., Ammar, A. A. A., Negm, S. H., Kawady, N. A., Rezk, M. M. (2024). Optimum factors estimation during uranium bioleaching process from three different samples using two different fungal strains. *Radiat. Nucl. Appl.* 9, 119-134. doi.org/10.18576/jrna/090204

### Wang, Y., Li, G., [Dexin](http://www.wanfangdata.com.cn/details/detail.do?_type=perio&id=hgxb201205037), Ding, D., Hu, N., Deng, Q., Zhou, Z. (2012). Factors influencing leaching of uranium ore by organic acids from Aspergillus niger. *CIESC J.* 63, 1584-1591. doi.org/10.3969/j.issn.0438-1157.2012.05.037

Wang, Y-D., Li, G-Y., Ding, D-X., Zhou, Z-X., Deng, Q-W., Hu, N., Tan, Y., (2013). Uranium leaching using mixed organic acids produced by *Aspergillus niger*. *J. Radioanal. Nucl. Chem*. 298, 769-773. doi.org/10.1007/s10967-013-2664-y

Wang, Y.-D., Li, G.-Y., Ding, D.-X., Zhang, Z.-Y., Chen, J., Hu, N., Li, L. (2015). Column leaching of uranium ore with fungal metabolic products and uranium recovery by ion exchange. *J. Radioanal. Nucl. Chem*. 304, 1139-1144. doi.10.1007/s10967-015-3957-0
